# Clinical Significance of Markers of Collagen Metabolism in Rheumatic Mitral Valve Disease

**DOI:** 10.1371/journal.pone.0090527

**Published:** 2014-03-06

**Authors:** Tanima Banerjee, Somaditya Mukherjee, Sudip Ghosh, Monodeep Biswas, Santanu Dutta, Sanjib Pattari, Shelly Chatterjee, Arun Bandyopadhyay

**Affiliations:** 1 Department of Cell Biology and Physiology, CSIR-Indian Institute of Chemical Biology, Kolkata, WB, India; 2 Department of Cardiology, Geisinger Community Medical Center and Wright Center for Graduate Medical Education, Scranton, Pennsylvania, United States of America; 3 Department of Cardio-thoracic and Vascular Surgery, Institute of Post Graduate Medical Education and Research, SSKM Hospital, Kolkata, WB, India; 4 Department of Pathology, Rabindra Nath Tagore International Institute of Cardiac Sciences, Kolkata, WB, India; University of Louisville, United States of America

## Abstract

**Background:**

Rheumatic Heart Disease (RHD), a chronic acquired heart disorder results from Acute Rheumatic Fever. It is a major public health concern in developing countries. In RHD, mostly the valves get affected. The present study investigated whether extracellular matrix remodelling in rheumatic valve leads to altered levels of collagen metabolism markers and if such markers can be clinically used to diagnose or monitor disease progression.

**Methodology:**

This is a case control study comprising 118 subjects. It included 77 cases and 41 healthy controls. Cases were classified into two groups- Mitral Stenosis (MS) and Mitral Regurgitation (MR). Carboxy-terminal propeptide of type I procollagen (PICP), amino-terminal propeptide of type III procollagen (PIIINP), total Matrix Metalloproteinase-1(MMP-1) and Tissue Inhibitor of Metalloproteinase-1 (TIMP-1) were assessed. Histopathology studies were performed on excised mitral valve leaflets. A p value <0.05 was considered statistically significant.

**Results:**

Plasma PICP and PIIINP concentrations increased significantly (p<0.01) in MS and MR subjects compared to controls but decreased gradually over a one year period post mitral valve replacement (p<0.05). In MS, PICP level and MMP-1/TIMP-1 ratio strongly correlated with mitral valve area (r = −0.40; r = 0.49 respectively) and pulmonary artery systolic pressure (r = 0.49; r = −0.49 respectively); while in MR they correlated with left ventricular internal diastolic (r = 0.68; r = −0.48 respectively) and systolic diameters (r = 0.65; r = −0.55 respectively). Receiver operating characteristic curve analysis established PICP as a better marker (AUC = 0.95; 95% CI = 0.91−0.99; p<0.0001). A cut-off >459 ng/mL for PICP provided 91% sensitivity, 90% specificity and a likelihood ratio of 9 in diagnosing RHD. Histopathology analysis revealed inflammation, scarring, neovascularisation and extensive leaflet fibrosis in diseased mitral valve.

**Conclusions:**

Levels of collagen metabolism markers correlated with echocardiographic parameters for RHD diagnosis.

## Introduction

Rheumatic Heart Disease (RHD) is a chronic acquired disorder of heart. It is a major public health concern in Low and Middle Income Countries (LMICs) having a global prevalence of at least 15.6 million cases, with 282,000 new cases and demanding 233,000 deaths each year [1]. It results as a sequelae of Acute Rheumatic Fever (ARF), caused by autoimmune reactions following untreated *Streptococcus pyogenes* throat infection in genetically susceptible individuals [2]. Acute rheumatic fever is a common health problem in developing countries [3]. It is estimated that around 12 million people, mainly children, suffer from the disease worldwide [4]. It is predicted that RHD would continue to cause high mortality and morbidity in LMICs and hence requires improved diagnostic and treatment strategies for disease management [5]. Based on WHO Global Burden of Disease, RHD has been categorized as a Neglected Tropical Disease (NTD) [6]. In India, Rheumatic heart disease is responsible for 30–40% cardiovascular disease related hospital admissions [7].

Although ARF causes pancarditis, it primarily affects endocardium and repeated episodes of autoimmune reactions cause chronic inflammation and scarring in valvular apparatus. Mitral valve is most commonly affected followed by aortic valve. The tricuspid and pulmonary valves are usually less severely affected. RHD in mitral valve manifests most commonly as mitral stenosis followed by mitral regurgitation or combination of both producing hemodynamic burden on heart causing clinical features like dyspnoea, palpitation and heart failure [8]. Management involves medical and surgical interventions for symptom alleviation and periodic clinical monitoring usually with transthoracic echocardiogram. In fact for prevention of heart failure and improvement of symptoms surgery is the treatment of choice. However till date, no biochemical indicators are available for prediction, diagnosis and management of the disease.

Fibrillar collagens (types I and III) arethe major components of the myocardial extracellular matrix. These are synthesized and secreted by fibroblasts as procollagen precursors. During the maturation process, the amino (N) terminal and carboxy (C) terminal propeptides are cleaved to yield triple helical monomers. The carboxy-terminal propeptide of type I procollagen (PICP) is released into the bloodstream during the synthesis of type I collagen. Therefore, PICP is considered to be a marker of type I collagen synthesis. Similarly amino terminal propeptide of type III collagen (PIIINP) is released into circulation during type III collagen synthesis. Fibrillar collagens are substrates for matrix metalloproteinases (MMPs), an endogenous family of zinc dependent enzymes which are responsible for extracellular matrix remodelling with tissue destruction under various pathophysiological conditions. Among them, MMP-1 has the highest affinity for fibrillar collagens. It is mainly secreted by fibroblasts and is able to initiate type I collagenolysis. Active form of MMP-1 can be regulated by its interaction with tissue inhibitor of matrix metalloproteinase-1 (TIMP-1), also expressed in the fibroblasts [9]. Marginal increase in serum PICP has been reported in various heart diseases like hypertensive heart disease [10], [11] cardiomyopathy and heart failure [12], [13]. But clinical significance of collagen metabolism markers, produced by structural remodelling of ECM in rheumatic valve has not yet been explored.

## Methods

### Study Population

The study population consisted of 118 subjects aged between 15 and 65 years. Rheumatic heart disease subjects who visited Cardio-thoracic and vascular surgery (CTVS) department of the Institute of Post Graduate Medical Education and Research- Seth Sukhlal Karnani Memorial Hospital (IPGME&R-SSKM), Kolkata, India from January 2010 to July 2012 were recruited into the study as cases.

Blood was collected from 72 RHD subjects in their chronic stage before surgical intervention (Pre-op) and out of them, 8 subjects were included again after valve replacement surgery (Post-op). In addition, for random validation, 5 post-op subjects were also included whose post-operative echocardiographic analysis could not be performed (Figure S1; Tables S1, S2 in File S1). Subjects with diabetes, hypertension, renal failure, metabolic bone disease, liver failure, severe systemic illness, rheumatoid arthritis or any organic heart disease except rheumatic heart disease were excluded from the studyafter careful clinical examination and relevant investigations. Patients with features of overt heart failure (LVEF<50%) were also not included.

Forty one healthy individuals with no evidence or family history of cardiac illness or past history of rheumatic fever or any medication use were selected as controls and underwent the same clinical examination and investigation protocols. All control subjects included in the study were normotensive, had normal cardiac function and lipid profile and also had normal fasting blood glucose levels. Most of the subjects who were recruited earlier have again been included in this study for comparing the different markers of collagen metabolism in mitral stenosis and regurgitation respectively which was not shown previously [14].

### Echocardiography

Two-dimensional transthoracic echocardiography with targeted M-mode and Doppler were performed using commercially available systems by trained cardiologists.Echocardiographic diagnosis of RHD conformed to the recommendation of the 2012 World Heart Federation criteria [15]. All measurements were made by blinded observers and mean of the three readings were recorded. Left atrial dimension and left ventricular dimension both at systole and diastole were recorded. Mitral valve area (MVA) was measured by planimetry and pressure half time (PHT) methods. The mean of these two readings were taken in subjects with mitral valve disease. Pulmonary artery systolic pressure (PASP) was also measured in these subjects. Ejection fraction was calculated by Simpson's method.None of the subjects had left ventricular systolic dysfunction as defined by an ejection fraction ≤50%. Doppler measurements of pressure gradients across valves was analyzed to assess the haemodynamic severity of lesions [15]. To assess LV hypertrophy, LV mass was calculated following the recommendations of the American Society of Echocardiography and European Association of Echocardiography using the Devereux formula: 0.89 (1.04 [LV internal diameter + posterior wall thickness + interventricular septal thickness]^3^^_^ [LV internal diameter]^3^) + 0.6 g. Relativewall thickness was estimated using the formula 2×posterior wall thickness/LV internal diameter in diastole [16]. RHD subjects were classified as Predominantly Mitral Stenosis (MS) with or without mild or moderate stenotic or regurgitant lesions (e.g; Aortic Stenosis, Aortic Regurgitation etc.) and Predominantly Mitral Regurgitation (MR) with mild or moderate other lesions [17].

### Clinical Examination

Clinical examination was performed by trained physicians and the data recorded in a structured pro forma. The patients' symptoms were recorded as per the New York Heart Association (NYHA) functional class. Systolic blood pressure (SBP) and diastolic blood pressure (DBP) were measured using standard cuff equipment at the clinic. Pulse rate including any irregularity was noted. Most recent electrocardiograms (ECGs) were reviewed. Cardio-thoracic ratio was measured from the chest skiagram performed in the subjects.

### Biochemical Determinations

Peripheral venous blood samples were drawn from healthy and chronic RHD subjects during clinical assessment and immediately subjected to plasma isolation. Each sample was centrifuged for 10 minutes at 4°C. The plasma was then separated into aliquots and stored at −80°C before analysis. Plasma concentrations of PICP (Takara Bio Inc. Shiga, Japan), PIIINP (Uscn Life Science Inc. Wuhan, P.R. China), total MMP-1 (AnaSpec, San Jose, CA, USA) and total TIMP-1 (R&D Systems, Minneapolis, MN, USA) were determined by enzyme immunoassay (EIA) using commercial assay systemsas per the manufacturer's instructions. Duplicate measurements were taken and the results averaged.The intra-assay and inter-assay variations for determining PICP concentrations were 6% and 5% respectively. The inter-assay and intra-assay coefficients of variation were <12% and <10% for MMP-1 and 4.9% and 3.9% for TIMP-1,respectively.Due to technical limitations, total MMP-1 and TIMP-1 were assessed in 17 controls and 42 subjects, PIIINP was measured in 34 controls and 63subjects and PICP was measured in 41 controls and 66 subjects.

### Histopathology

Anterior leaflets of mitral valvetissue samples, from subjects undergoing valve replacement and from post-mortem control subjects without any known history of cardiovascular disease were collected and fixed in 10% formalin. Samples were then embedded in paraffin and 5 µm thick sections were prepared according to standard procedure. Tissue sections were either stained with hematoxylin - eosin (HE stain, Sigma Chemical Co., St Louis, MO, USA) as described earlier [18] or with Masson Trichrome to examine fibrosis. Sections were examined under an Olympus BX51 (Olympus Corporation, Tokyo, Japan) microscope and images were captured with a digital camera attached to it. Picrosirius red -0.1% Sirius red F3B solution in picric acid (Direct Red 80; Sigma Chemical Co., St. Louis, MO, USA) staining was also performed in valve leaflets to examine the collagen content and alignment and infer type of collagen. Stained sections therefore were observed using a binocular polarized light microscope (Nikon Eclipse binocular polarising microscope, LV 100 POL, Japan) with the same exposure time for each section. Under polarized light, birefringence is specific for collagen where red colour shows fibrillar type I collagen and yellow green colour indicates reticular type III collagen [19].

### Immunohistochemistry

Immunohistochemical staining of formalin fixed paraffin embedded tissue sections were performed following the protocol described in [20], [21]. First sections were deparaffinized in xylene twice for 2 min each, followed by serial hydration steps in alcohol: 100% for 3 min, 95% for 1 min, 70% for 1 min. Sections were then sequentially rinsed in running tap water and phosphate buffer saline (PBS) for 2 min each, followed by antigen retrieval in citrate buffer. Tissue areas were marked and the sections were washed with PBS in 0.2% Triton X-100, thrice for 5 min, followed by blocking in 5% BSA at room temperature for 2 hours. Staining was performed by incubating the sections with anti-collagen type I primary antibody (C-2456), (Sigma Chemical Co., St. Louis, MO, USA) overnight at 4°C followed by washing three times with PBS. Secondary antibody conjugated with Alexa Fluor, (Molecular Probes, Life Technologies, Grand Island, NY, USA) was incubated at room temperature for 1 hour followed by washing thrice with PBS. Finally coverslips were mounted with Prolong Gold anti-fade reagent (Molecular Probes, Life Technologies, Grand Island, NY, USA) and visualized under a laser scanning confocal system (Leica Microsystem, GmbH, Wetzlar, Germany).

### Statistical Analysis

Data are expressed as mean ± standard error of mean (SEM) for continuous variables, while frequencies define categorical variables. Using Microcal Origin 8.0 software (Origin Lab, Northampton,USA) normal distribution of all continuous variables was tested using the 1-sample Kolmogorov-Smirnov test. Categorical variables were analysed by Chi square (χ^2^) or Fisher's exact test. Differences between the two groups of RHD subjects were tested by Student's *t-* test for unpaired and paired data. One way Analysis of Variance (ANOVA) followed by Post-hoc tests was used to compare biomarker levels among controls and the two groups of subjects. Non-parametric tests were used when required.The correlation between quantitative normal variables was analysed by linear regression using Microsoft Excel 2010. Receiver operating characteristics (ROC) curves, prepared using GraphPad Prism software 5.30 (GraphPad, San Diego,California,USA) assessed the overall performance of plasma biomarkers. A two-tailed p value ≤0.05 was considered statistically significant.

### Ethics Statement

The study protocol which conformed to the principles of the Declaration of Helsinki for biomedical research, was approved by the Institutional Review Boards of the Indian Institute of Chemical Biology, Kolkata and IPGME&R-SSKM, Kolkata, India. Written informed consent was obtained from all subjects before participation in the study.

## Results

### Baseline Characteristics Analysis

Baseline characteristics of subjects recruited into the study are presented in Tables 1 and 2 respectively. Table 1 shows that almost an equal percentage of males and females comprised the control group. Heart rate, Diastolic Blood Pressure (DBP) and all the echocardiographic parameters except Left Ventricular Posterior Wall (LVPW) and Interventricularseptal Diameter (IVSD) were significantly different (p<0.05) between the control and patient groups. As shown in Table 2, about 63% of RHD subjects (45 of 72 subjects) were female. Around 52% of MS subjects were females while in the MR group about 77% of the subjects were females.The clinical and demographic variables were comparable between the RHD subgroups except for age. About 69% patients had a previous history of Acute Rheumatic Fever.39% RHD subjects were in NYHA class I,40% belonged to NYHA class II,15% belonged to class III and the remaining 6% in class IV. All control subjects belonged to NYHA class I (Table 1). None of the controls under study had any history of medication intake during the course of study. On the other hand, 74%, 25% and 65% of the RHD subjects were taking diuretics (furosemide, torsemide), spironolactone and digoxin respectively. 15% were on beta blockers, 19% had history of intake of ACE inhibitors and 43% of subjects were on anticoagulant therapy (Table 2). All RHD subjects included in the study were under rigorous penicillin prophylaxis to prevent recurrent episodes of acute rheumatic fever.

**Table 1:** Baseline Characteristics.

Variables	Control (n = 41)	Case (n = 72)	p value
Age (years)	32.34±1.34	34.26±1.50	0.64
Male/Female	26/25	27/45	0.15
**Clinical Presentation data**			
Pulse (bpm)	75.32±1.87	81.25±1.56	0.03
SBP (mm Hg)	117.22±1.90	112.86±2.01	0.15
DBP (mmHg)	79.88±1.40	72.86±1.45	0.002
NYHA class			
I	41(100%)	28(38.89%)	<0.0001
II	0	29(40.28%)	NA
III	0	11(15.28%)	NA
IV	0	4(5.56%)	NA
**Echocardiographic parameters (Normal range)**			
LA (20–40 mm)	30.41±0.53	54.25±1.48(n = 65)	<0.0001
LVIDd (35–56 mm)	42.80±0.76	51.77±1.19	<0.0001
LVIDs (24–42 mm)	27.39±0.54	34.70±0.82	<0.0001
LVPW (6–11 mm)	8.61±0.13	8.81±0.19(n = 70)	0.59
IVSD (6–11 mm)	8.71±0.14	9.07±0.21(n = 70)	0.29
EF (%)	66.95±0.59	58.60±0.82	<0.0001
Fractional shortening (%)	35.79±0.94	32.64±0.83	0.006
Relative Wall Thickness	0.41±0.01	0.36±0.01(n = 70)	0.0002
Left Ventricle Mass (Kg)	0.12±0.004	0.18±0.008(n = 70)	<0.0001

BPM beats per minute; DBP diastolic blood pressure; EF ejection fraction; IVSD inter-ventricular septal diameter; LA left atrium; LVIDd left ventricular internal diameter diastolic; LVIDs left ventricular internal diameter systolic; LVPW left ventricular posterior wall; n number of subjects; NA not applicable; NYHA New York Heart Association; SBP systolic blood pressure. Values indicate mean ± standard error of mean. p<0.05 considered significantly different between two groups.

**Table 2:** Baseline Patient Characteristics.

Variables	Predominantly MS (n = 42)	Predominantly MR (n = 30)	p value
Age(years)	37.55±1.81	29.67±2.35	0.009
Male/Female	20/22	7/23	0.04
Clinical Presentation data			
H/o Rheumatic Fever	29(69%)	20(66.67%)	1.00
Pulse (bpm)	79.38±2.09	83.87±2.30	0.16
SBP(mm Hg)	112.29±2.70	113.67±3.05	0.74
DBP(mmHg)	73.95±2.05	71.33±1.96	0.38
CT ratio(n-58)	0.56±0.02(n = 25)	0.63±0.04(n = 12)	0.14
Atrial fibrillation	15(35.71%)	7(23.33%)	0.31
Premature atrial complex	1	0	NA
**NYHA class**			
I	19(45.24%)	9(30%)	0.23
II	16(38.09%)	13(43.33%)	0.81
III	6(14.29%)	5(16.67%)	1.00
IV	1(2.38%)	3(10%)	0.30
Medicine use			
Diuretics	29(69.05%)	24(80%)	0.42
Spironolactone	9(21.43%)	9(30%)	0.42
ACE inhibitors	1(2.38%)	13(43.33%)	<0.0001
ARBs	0	0	NA
β blockers	8(19.05%)	3(10%)	0.34
Digoxin	27(64.29%)	20(66.67%)	1.00
Warfarin	21(50%)	10(33.33%)	0.23

Variables	Predominantly MS (n = 42)	Predominantly MR (n = 30)	p value
**Echocardiographic parameters (Normal range)**			
LA (20–40 mm)	51.56±1.36(n = 37)	57.81±2.81(n = 28)	0.04
LVIDd (35–56 mm)	45.89±1.04	59.79±1.58	<0.0001
LVIDs (24–42 mm)	31.83±0.82	38.92±1.27	<0.0001
LVPW (6–11 mm)	8.73±0.28(n = 40)	8.91±0.25	0.63
IVSD (6–11 mm)	9.08±0.31(n = 40)	9.07±0.26	0.97
EF (%)	56.98±1.08	60.88±1.18	0.02
PASP (mm Hg)	57.85±3.83(n = 24)	55±5.15(n = 12)	0.66
MVA (sq.mm)	84±3(n = 40)	226±23(n = 11)	<0.0001
Fractional shortening(%)	31.13±0.94	34.76±1.44	0.03
Relative Wall Thickness	0.39±0.02(n = 40)	0.31±0.01	0.002
Left Ventricle Mass(Kg)	0.14±0.007(n = 40)	0.23±0.014	<0.0001

ACE angiotensin converting enzymes; ARB angiotensin receptor blocker; bpm beats per minute; CT cardio-thoracic; DBP diastolic blood pressure; EF ejection fraction; H/o history of; IVSD inter-ventricular septal diameter; LA left atrium; LVIDd left ventricular internal diameter diastolic; LVIDs left ventricular internal diameter systolic; LVPW left ventricular posterior wall; MVA mitral valve area; n number of subjects; NA not applicable; NYHA New York Heart Association; PASP pulmonary artery systolic pressure; SBP systolic blood pressure. Values indicate mean ± standard error of mean.

p<0.05 considered significantly different between two groups.

Among the echocardiographic parameters, Left atrial (LA) diameter, Mitral Valve Area (MVA), Left ventricular ejection fraction (EF), Left ventricular internal diameter in diastole (LVIDd), left ventricular internal diameter in systole (LVIDs), Left Ventricle (LV) Mass and Fractional Shortening (FS) were significantly higher in MR subjects (p<0.05) compared to MS subjects. Relative Wall Thickness (RWT) also differed between the two groups of RHD subjects (Table 2).

### Circulating markers of collagen metabolism are elevated in Rheumatic Mitral Valve Disease

**Figure 1:**
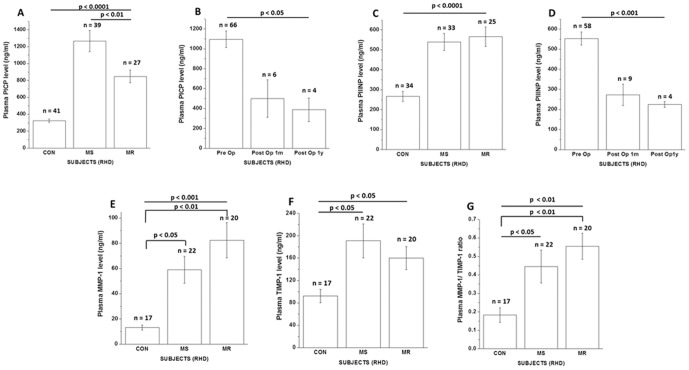
Plasma concentrations of circulating biomarkers of collagen turnover in normal, MS and MR subjects. (A) Mean plasma PICP in control, MS and MR subjects before (Pre Op) valve replacement surgery. (B) Progressive reduction in plasma PICP concentration one month and one year or above following mitral valve replacement. (C) Mean plasma PIIINP in control, MS and MR subjects before (Pre Op) valve replacement surgery. (D) Progressive decrease in plasma PIIINP concentration one month and one year or more after mitral valve replacement. (E) Mean plasma concentration of total MMP-1 in control, MS and MR subjects. (F) Mean TIMP-1 concentration in control, MS and MR subjects. (G) Plasma MMP-1/TIMP-1 ratio in control, MS and MR subjects.

Plasma PICP levels were significantly different among the three groups of subjects as assessed by one-way ANOVA (p<0.0001; Figure 1A). Post-hoc tests reveal that PICP levels were significantly (p<0.01) higher in MS (1265±125 ng/ml, n = 39) and MR (848±74 ng/ml, n = 27) subjects than controls (324±18 ng/ml, n = 41).Level of PICP also differed significantly between MS and MR subjects (p<0.01). Significant decrease in circulating PICP levels was observed one month (2.2 fold) and one year (3 fold) post mitral valve replacement surgery (p = 0.02; Figure 1B).

Figure 1C showed the distribution of plasma PIIINP levels in MS and MR patients along with control subjects. Patients with RHD showed higher PIIINP levels (p<0.0001). Plasma PIIINP concentration was significantly different (p<0.01) between MS (539±42 ng/ml, n = 33) and controls and between MR (565±48 ng/ml, n = 25) subjects and controls (266±24 ng/ml, n = 34) but not between MS and MR subjects. The extent of reduction in circulating PIIINP levels post mitral valve replacement surgery was also significant over a one month (2.1 fold) and a one year (2.6 fold) period (p<0.001; Figure 1D). Again, when paired comparisons were performed, PIIINP level (547±99 ng/ml) decreased significantly one month post -surgery (212±14 ng/ml, Δ pre -post  = 257%, n = 6, p<0.01; Figure S2).

Total plasma MMP-1 values showed a significantly different distribution across the groups (p = 0.0002). Compared to controls, total MMP- 1 level (13±1.9 ng/ml, n = 17) increased significantly in subjects with MS (59±10 ng/ml, n = 22, p<0.05) and MR (82±14 ng/ml, n = 20, p<0.01; Figure 1E). Total TIMP-1 levels varied significantly among the three groups (p = 0.02; Figure 1F). Compared to controls (92±12 ng/ml, n = 17), levels were significantly higher in MS (191±30 ng/ml, n = 22; p<0.05) but not in MR patients (160±21 ng/ml, n = 20). Also MMP-1/TIMP-1 ratio, an index of MMP-1 activity was significantly different among the three groups (p = 0.003; Figure 1G). The ratio increased in both MS and MR groups by about 2.5 fold and 3.1 fold respectively, compared to controls. There was no significant difference in the levels of collagen metabolism markers between male and female subjects in both MS and MR categories (Table S3 in File S1). It was also observed that TIMP-1 and PICP levels were significantly higher in MS and MR subjects respectively with atrial fibrillation (p<0.05; Table S4 in File S1).

### Assessment of diagnostic accuracy of indices of collagen metabolism

**Figure 2:**
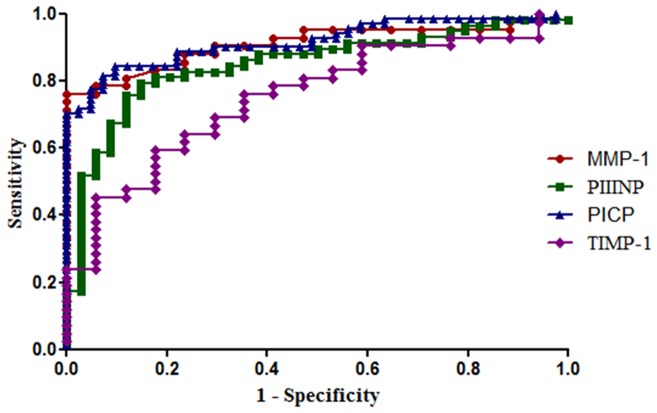
Receiver operating characteristics curve for biomarkers in rheumatic heart disease subjects.

As shown in Figure 2, ROC curve analysis demonstrates plasma PICP, total MMP-1 and PIIINP as significant predictors of rheumatic heart disease (Table 3).PICP performed better than MMP-1, PIIINP or TIMP-1 with AUC of 0.95 (Table 3). Overall, the cut off value of PICP showed the best sensitivity and specificity for predicting valve fibrosis (Table 3). Thus the likelihood of presenting severe mitral valve disease of rheumatic originwas 9.32 times higher for subjects with PICP values >459 ng/ml, 4.59 times higher for subjects with PIIINP values >351 ng/ml, 4.72 times higher for subjects with MMP-1 values >21.8 ng/ml and 2.35 times higher for subjects with TIMP-1 values >105 ng/ml (Table 3). The sensitivity of PICP was 92% in Mitral Stenosis (AUC = 0.97) and 89% in Mitral Regurgitation (AUC = 0.91). The sensitivity of PIIINP was 82% in Mitral Stenosis(AUC = 0.84) and 80% in Mitral Regurgitation (AUC = 0.86). However the sensitivity of MMP-1 was found to be 90% in MR (AUC = 0.97) while it was about 77% in Mitral Stenosis (AUC = 0.85). The sensitivities of TIMP-1 were comparable between Mitral Stenosis and Mitral Regurgitation patients (AUC = 0.75 in both) (Tables 4 and 5 respectively.)

**Table 3:** Overall Performance of Different Parameters According to ROC Curves for Prediction of RHD.

Biomarkers	Cut Off	Sensitivity (%)	Specificity (%)	AUC	p value	LR	95% CI	PPV	NPV
PICP (ng/ml)	>459	90.9	90.2	0.95	<0.0001	9.32	0.91 to 0.99	0.94	0.86
PIIINP (ng/ml)	>351	81	82.3	0.85	<0.0001	4.59	0.77 to 0.93	0.89	0.72
MMP-1 (ng/ml)	>21.8	83.3	82.3	0.91	<0.0001	4.72	0.84 to 0.98	0.95	0.68
TIMP-1 (ng/ml)	>105	69	70.6	0.75	0.003	2.35	0.62 to 0.88	0.89	0.72

p <0.05 considered significantly different.

AUC, area under curve; CI, confidence interval; LR, likelihood ratio; MMP-1, matrix metalloproteinase -1; NPV, negative predictive value; PICP, carboxy terminal propeptide of type I collagen; PIIINP, amino terminal propeptide of type III collagen; PPV, positive predictive value; TIMP-1, tissue inhibitor of matrix metalloproteinase-1.

**Table 4:** Overall Performance of Different Parameters According to ROC Curves for Prediction of Mitral Stenosis.

Biomarkers	Cut Off	Sensitivity (%)	Specificity (%)	AUC	p value	LR	95% CI	PPV	NPV
PICP (ng/ml)	>489.3	92.3	92.7	0.97	<0.0001	12.6	0.95 to 1.00	0.92	0.93
PIIINP (ng/ml)	>351.4	81.8	82.4	0.84	<0.0001	4.64	0.73 to 0.94	0.82	0.82
MMP-1 (ng/ml)	>19.94	77.3	76.5	0.85	0.0002	3.28	0.73 to 0.98	0.81	0.72
TIMP-1 (ng/ml)	>108	68.2	70.6	0.75	0.007	2.32	0.60 to 0.91	0.75	0.63

p<0.05 considered significantly different.

AUC, area under curve; CI, confidence interval; LR, likelihood ratio; MMP-1, matrix metalloproteinase -1; NPV, negative predictive value; PICP, carboxy terminal propeptide of type I collagen; PIIINP, amino terminal propeptide of type III collagen; PPV, positive predictive value; TIMP-1, tissue inhibitor of matrix metalloproteinase-1.

**Table 5:** Overall Performance of Different Parameters According to ROC Curves for Prediction of Mitral Regurgitation.

Biomarkers	Cut Off	Sensitivity (%)	Specificity (%)	AUC	p value	LR	95% CI	PPV	NPV
PICP (ng/ml)	>457	88.9	87.8	0.91	<0.0001	7.29	0.82 to 0.99	0.83	0.91
PIIINP (ng/ml)	>341.7	80	79.4	0.86	<0.0001	3.89	0.76 to 0.96	0.74	0.84
MMP-1 (ng/ml)	>23.49	90	88.24	0.97	<0.0001	7.65	0.93 to 1.02	0.9	0.88
TIMP-1 (ng/ml)	>105.1	70	70.6	0.75	0.009	2.38	0.59 to 0.91	0.82	0.67

p<0.05 considered significantly different.

AUC, area under curve; CI, confidence interval; LR, likelihood ratio; MMP-1, matrix metalloproteinase -1; NPV, negative predictive value; PICP, carboxy terminal propeptide of type I collagen; PIIINP, amino terminal propeptide of type III collagen; PPV, positive predictive value; TIMP-1, tissue inhibitor of matrix metalloproteinase-1.

### Relationship of Collagen Metabolism Markers with severity determinants in Predominantly Mitral Stenosis

**Figure 3:**
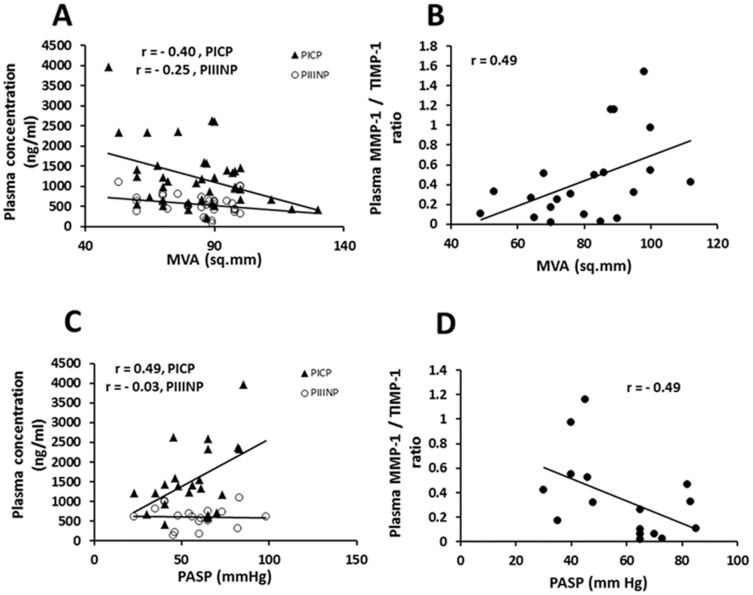
Relationship between plasma markers of collagen metabolism and severity of rheumatic mitral stenosis. (A) Inverse correlations of plasma PICP (y = −17.241x+2654.1; p = 0.01) and PIIINP (y = −4.6576x+938.36; p = 0.15) concentration with mitral valve area(MVA). (B) Direct correlation (y = 0.0127x−0.582; p = 0.03) between plasma MMP-1/TIMP-1 ratio and MVA. (C) Direct correlation of plasma PICP (y = 24.155x+186.83;p = 0.02) and almost no correlation of plasma PIIINP (y = −0.4083+634.78;p = 0.91) with pulmonary artery systolic pressure (PASP). (D) Inverse correlation (y = −0.0091x+0.8791; p = 0.05) between plasma MMP-1/TIMP-1 ratio and PASP.

In MS patients, plasma PICP showed a strong inverse correlation with MVA while MMP-1/TIMP-1 ratio showed a strong positive association with it (Figure 3A, 3B). Plasma PICP levels also correlated positively with PASP while MMP-1/TIMP-1 ratio correlated inversely (Figures 3C, 3D). Plasma PICP showed inverse associations with LVPW and IVSD respectively while MMP-1/TIMP-1 ratio inversely correlated with EF and FS and positively with LVIDs (Table S5 in File S1) in this group. Total MMP-1 levels correlated positively with LVIDs and inversely with EF and FS. PIIINP levels were found to positively associate with EF (Table S5 in File S1) and weakly correlated with MVA or PASP (Figures 3A, 3C).

### Relationship of Collagen Metabolism Markers with severity determinants in Predominantly Mitral Regurgitation

**Figure 4:**
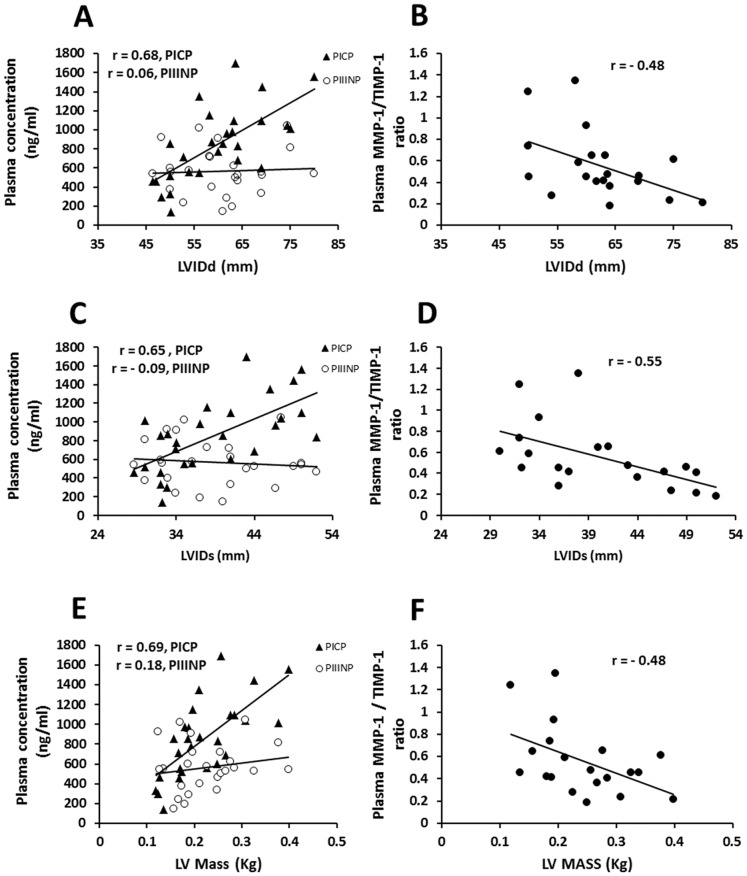
Relationship between plasma PICP levels and hemodynamic parameters in rheumatic mitral regurgitation. (A) Direct correlation of plasma PICP (y = 28.83x−875.54;p = 0.0001) and no correlation of plasma PIIINP (y = 1.5469x+471.72; p = 0.79) with left ventricular diameter at diastole (LVIDd). (B) Inverse correlation (y = −0.0181x+1.6878; p = 0.04) between plasma MMP-1/TIMP-1 ratio and ventricular diameter at diastole (LVIDd). (C) Direct correlation of plasma PICP (y = 34.933x−506.05; p = 0.0002) and no correlation of plasma PIIINP (y = −3.3609x+697.11; p = 0.64) with left ventricular internal diameter at systole (LVIDs). (D) Inverse correlation(y = −0.0241x+1.523;p = 0.01) between plasma MMP-1/TIMP-1 ratioand LVIDs. (E) Direct correlation of plasma PICP (y = 3620.7x+53.656; p<0.0001) and weak correlation of plasma PIIINP (y = 593.3x+430.94; p = 0.39) with left ventricular mass (LVM). (F) Inverse correlation (y = −1.9248x+1.0243;p = 0.03) between plasma MMP-1/TIMP-1 ratio and LVM.

In MR patients circulating PICP strongly associated with LVIDd, LVIDs and LV mass while MMP-1/TIMP-1 ratio showed strong inverse relationship with the above parameters (Figures 4A to 4F). Plasma PICP correlated positively with LA diameter and negatively with relative wall thickness while circulating PIIINP levels showed a strong positive association with PASP in this group (Table S6 in File S1) and weak correlation with LVIDs. Plasma TIMP-1 levels however, did not correlate significantly with any of the echocardiographic parameters in MS and MR.

### Histopathology Analysis

**Figure 5:**
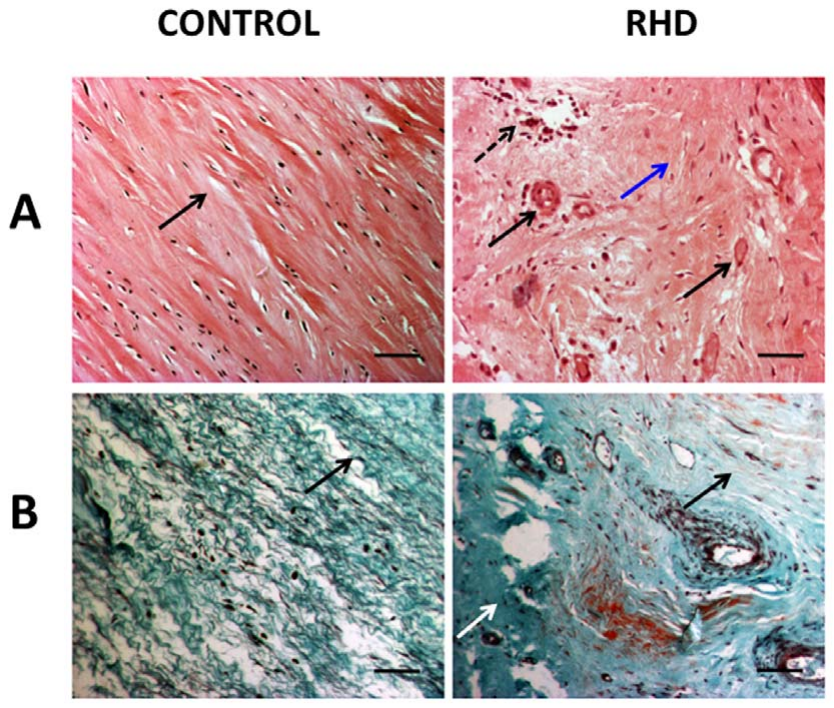
Histopathology of heart valve. (A) Representative images (100× magnification) of hematoxylin-eosin stained sections of 1 normal heart valve (control) and 3 rheumatic valve samples (RHD). Rheumatic mitral valve tissue section shows abundance of inflammatory cells (arrow head), fibrosis (blue arrow) and neovascularisation (black arrow). Normal mitral valve section shows wavy arrangement of collagen fibres (black arrow). Scale bar represents 50 µm. (B) Representative images (100×) of Masson's trichrome stained cross sections showing dense collagen deposition (arrow) in mitral valve tissue of RHD patient compared to loose parallel pattern of collagen (arrow) in normal heart valve. Scale bar represents 50 µM.

Gross examination of HE stained mitral valve sections from the RHD subjects showed thickening of valve leaflets with irregular margins. Microscopic examination showed enhanced degree of fibrosis and neovascularisation (arrows marked in Figure 5A). Focal perivascular mild infiltration of lymphocytes and plasma cells were prominent, thus confirming inflammatory aetiology in those subjects. In contrast, normal valve was composed of loose collagen tissue with absence of blood vessels and inflammatory cells (Figure 5A).

**Figure 6:**
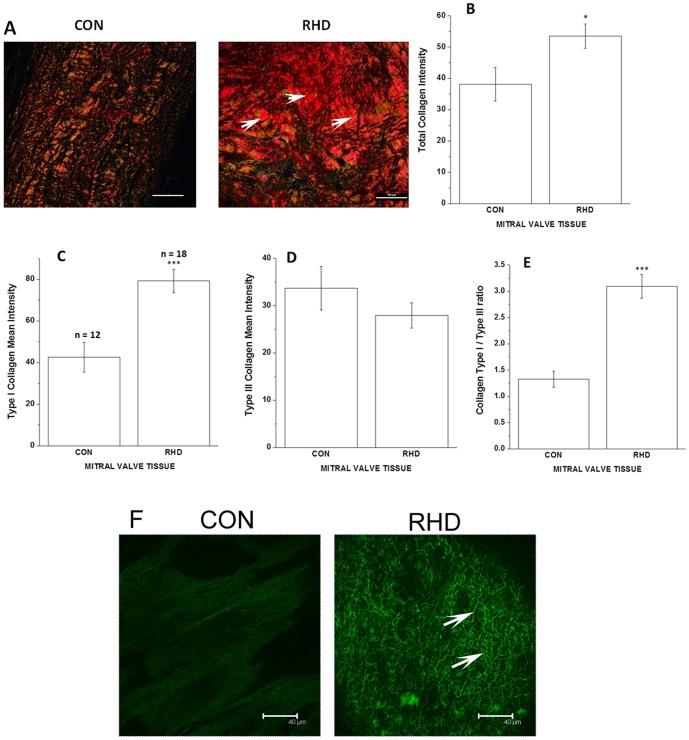
Assessment of collagen deposition by picrosirius red staining and immunostaining methods. (A) Representative images (20× magnification) of picrosirius red stained sections of 1 normal heart valve (control) and 3 rheumatic mitral valve samples (RHD). Stained sections were observed using a binocular polarized light microscope. Under polarized light, birefringence is specific for collagen where red colour shows fibrillar type I collagen and yellow green colour indicates reticular type III collagen. Arrow indicates scattered deposition of collagen type I in diseased valve Scale bar represents 100 µm. (B) Total collagen intensity in control vs. RHD mitral valve tissue sections. (C) Type I collagen mean intensity in control vs. RHD mitral valve cross sections. (D) Type III collagen mean intensity in control vs. RHD mitral valve cross sections. (E) Ratio of Type I to Type III collagen in control vs. RHD mitral valve sections.(F) Representative images of immuno stained sections of 1 normal heart valve (control) and 3 rheumatic mitral valve samples (RHD) showing (arrow marked) collagen type 1 deposition. Scale bar represents 45 µm. *p<0.05 vs. control,***p<0.0001 vs. control. Here “n” denotes total number of tissue sections.

Masson trichrome staining shows collagen deposition in valve leaflets of RHD subjects and controls. Normal mitral valve shows loose parallel arrangement of collagen fibres whereas diseased valve shows dense and extensive collagen fibres, predominantly arranged in random pattern (Figure 5B). A representative picture illustrating the relative abundance of collagens type I (red colour) and type III (green colour) in the mitral valve leaflets of the subjects under polarized light is shown in Figure 6A. The abnormal accumulation of fibrous tissue was seen as a scattered deposition of collagen type I (red deposition) in diseased valve leaflets leading to a marked disarray of the ECM architecture. The extent of total collagen (p<0.05), collagen type I deposition as well as collagen type I/type III ratio was higher (p<0.0001) in the rheumatic mitral valve than normal valve (Figures 6B–6E). This is further supported by immunofluorescence analysis of mitral valve leaflets using anti-collagen I antibody which clearly showed increased and scattered collagen I deposition in RHD subjects (Figure 6F).

## Discussion

In present perspective, 2D Doppler echocardiography has proved to be an essential diagnostic tool not only in clinically determined RHD but also in subclinical RHD [22], [23]. In this form of RHD, features of valve damage like annular dilatation, prolapsed leaflet, trivial-mild regurgitation could be detected on echocardiography without clinical symptoms or signs of RHD. However, role of echocardiography to confirm diagnosis of subclinical RHD still remains controversial, especially in a developing country like India. There is also a considerable degree of overlap between normal and pathological findings on echocardiography in detecting subclinical RHD. In India where burden of rheumatic fever cases is large and highly skilled echo-Doppler facilities are available only in limited number of centres, accurate diagnosis of RHD would be difficult [15], [24], [25]. However, the acute management of such patients and the duration of secondary prophylaxis would not change significantly, even if a diagnosis of subclinical carditis were made. In the present study we have mainly focused on early cases of established RHD where timely determination of severity and prognostication may alter therapeutic strategies, and prevent further progression to advanced stages with low cardiac performance.

Mitral valve leaflets are composed of 74% type I, 24% of type III and 2% of type V collagen. Fibrous tissues intermixed with elastic fibres form the extracellular matrix (ECM) of valvular apparatus covered by a layer of endothelial cells [26]. Fibrosis is the hallmark of structural remodelling, resulting from chronic inflammatory rheumatic process in valvular apparatus. Fibrosis results from extensive deposition of collagen induced by mechanical overload combined with the action of other pro fibrotic factors or tissue damage.

A significantly raised plasma concentration of PICP observed in both MS (377%) and MR (245%) could be explained by anincreased production and deposition of collagen over heart valves by myofibroblasts [27], [28]. During inflammatory process like chronic RHD, cytokines are released which lead to differentiation of valvular interstitial cells (VICs) to activated myofibroblasts [27]. VICs are the most abundant cells in valve leaflets which produce various kinds of extracellular matrix (ECM) proteins including collagen. They also produce ECM degrading enzymes like MMPs. The above finding is also supported by collagen birefringence under polarized light whichindicates a 186% increase in Type I collagen in diseased valve tissue than control. Earlier report have shown increased deposition of collagen on excised RHD valves [29]. Elevated plasma level of PIIINP in MS (200%) and MR (212%) in the present study gets supported by previous studies which have shown that activated myofibroblasts result in fibrosis through increased production of both type I and type III collagen [30]. Active role of VICs in producing MMPs like collagenases (MMP-1, MMP-13) and gelatinases (MMP-2, MMP-9) have also been reported [31]. High level of total MMP-1 in MS (446%) and MR (624%) in our study indicated that in addition to increased level of markers of synthesis, collagen degrading enzymes are also raised in RHD.

Rheumatic mitral stenosis occurs due to thickening of the mitral valve leaflets and fusion of commissures and chordae tendineae. Earlier it was reported that PICP may determine clinical severity in mitral stenosis since it inversely correlated with MVA and positively correlated with PASP in mitral stenosis with mild or no mitral regurgitation [14]. But the association of other markers of ECM remodelling like PIIINP and MMP-1/TIMP-1 ratio with the above parameters were not dealt with previously. Besides, the relationship between biochemical markers of collagen metabolism and haemodynamic parameters in mitral regurgitation remained to be established in greater detail. Therefore in this study, previous data of PICP of some of the individuals have been included to compare its performance with PIIINP and collagenolytic markers. However unlike PICP, PIIINP showed weak correlation with MVA and PASP in MS. Since PIIINP is not cleaved completely during the conversion of procollagen type III into collagen type III and part of it is released during fiber degradation, PIIINP can be considered to be a marker of both collagen synthesis and degradation. Hence, it is weakly associated with echocardiographic parameters in both MS and MR. In MS, correlation of MMP-1/TIMP-1 ratio with MVA and PASP followed an opposite trend to that shown by PICP, indicating that collagen synthesis on mitral valve is more favoured compared to degradation with disease progression in MS. Besides valvular apparatus, atrial fibrosis may also have some contribution to altered levels of collagen metabolism markers in MS subjects. Such atrial fibrosis is supposed to be responsible for atrial fibrillation found in rheumatic mitral stenosis [32].

In rheumatic mitral regurgitation primary morphological change occur in mitral valvular apparatus which leads to secondary remodelling in LV. This includes post inflammatory thickening and fusion of the mitral valve leaflets at the commissures along with scar retraction producing an eccentrically located funnel shaped orifice. It results in reduced valve motion in both systole and diastole. Such restrictive mechanism of regurgitation is unique to rheumatic heart disease and differs from degenerative valvular regurgitation [26]. Left ventricular (LV) dilatation and eccentric hypertrophy in mitral regurgitation are secondary remodelling due to haemo-dynamic volume overload on LV produced by primary morphological change in the mitral valve in RHD. In compensated phase of mitral regurgitation, with increase in end diastolic volume, there is a compensatory dilatation of ventricle. This eccentric hypertrophy initially helps to maintain high stroke volume in compensated phase. But in decompensated phase, progressive ventricular dilatation causes increase in wall stress with tissue damage and contractile dysfunction [33], [34].

Ultrastructurally in volume overload states, sarcomere replication in series causes myocyte lengthening (Frank-Starling mechanism) resulting in ventricular dilatation. Besides, dilatation or eccentric hypertrophy in mitral regurgitation is also contributed by alteration in collagen cross linking and break down of collagen weave by MMP activation. This is entirely different from pressure overload states where an extensive production and deposition of collagen occurs in the ventricle itself [35]. Therefore it is likely that in spite of progressive ventricular dilatation due to collagen weave degradation (secondary remodelling), there is an elevation in plasma level of PICP in MR and it also directly associates with LVIDd, LVIDs and LV mass. It may be due to extensive fibrosis on valvular apparatus (primary event) that occurs concomitantly and progressively with ventricular dilatation. However elevation in plasma PICP concentration is somewhat compromised in MR particularlysince degradation activity is increased in MR. This is evident from a significant increase in MMP-1 level in MR compared to MS subjects as mentioned earlier.This also explains an inverse correlation of MMP-1/TIMP-1 ratio with the above LV parameters in MR.

Significant decrease in plasma PICP and PIIINP levels following mitral valve replacement after one month and one year strongly suggest contribution of mitral valve apparatus in altered levels of collagen metabolism markers in RHD. Thus fibrotic state of the mitral valve precedes the occurrence of myocardial dysfunction and overt heart disease. This information might be helpful for the development of diagnostic and therapeutic strategies by targeting structural remodelling of the heart valves in RHD.

Though in our present study we have shown a 141% higher deposition of total interstitial collagen on rheumatic mitral valve, due to technical limitations, histological alterations and collagen distribution in other parts of cardiac tissue could not be shown. It would be interesting if some controls with same hemodynamic status (heart valve disease) but not related to RHD were considered in order to better appreciate the specificity of our findings. Also the presence of a larger multi-centric population would have helped to validate the current findings.

Adequate information and improved data on chronic diseases of developing nations such as rheumatic heart disease are lacking presumably because limited research activities are carried out worldwide on such diseases. This may be attributed to the rare incidence of RHD in many of the developed nations around the world today. Hence a disease like RHD that still remains a public health burden in LMICs lacks sufficient attention globally. Antibiotic prophylaxis is recommended as the most effective approach for control of recurrent attacks of ARF; yet most of the cases progress to chronic RHD. Echocardiography has an established role not only in diagnosis but also determines severity of RHD. However it is costly and only available in limited tertiary health care facilities. As a result due to lack of monitoring most of them progress to advanced RHD with low ejection fraction. Sometimes other than conservative medical treatment, even surgery may not be considered a possible option in them. So there is a need for a biomarker that would correlate with the echocardiographic progression of valve damage and would decide therapeutic interventions much earlier. Result of our present study based on biomarkers of collagen metabolism is a step forward towards that goal. As our study has shown that increased fibrosis correlates with echo parameters of advanced disease, attempts could be made for development of cardiac specific antifibrotic medication that may prevent permanent morphological damage of heart valves. However it may be mentioned that the target population for carrying out active surveillance programme in endemic regions with the help of collagen metabolism markers needs to be defined clearly in further studies as collagen is a ubiquitous protein and degradation products may be detectable in a variety of other conditions. Moreover procuring and preserving blood samples and rapid detection in field may be cumbersome with available technologies. Finally, undertaking cross country research collaboration initiatives involving multiple centres would help to provide new insights for better understading and management of the disease.

## Supporting Information

Figure S1 **Flow Chart of Study Design.**
(TIF)

Figure S2 **Comparison of plasma PIIINP level of the same RHD subjects (n = 6) before surgery and one month after mitral valve replacement.**
(TIF)

File S1 Table S1. Characteristics of randomly collected post-operative RHD subjects. Table S2. Preoperative Echocardiographic Characteristics of randomly collected post-operative RHD subjects. Table S3 Level of collagen metabolism markers in male and female subjects with Mitral Stenosis and Mitral Regurgitation lesions. Table S4 Level of collagen metabolism markers in subjects with and without Atrial Fibrillation in Mitral Stenosis and Mitral Regurgitation groups. Table S5 Association of collagen metabolism markers with continuously distributed variables in subjects with Mitral Stenosis. Table S6. Association of collagen metabolism markers with continuously distributed variables in subjects with Mitral Regurgitation.
(DOC)
