# Supplementary figures and images for: Clinical Significance of Markers of Collagen Metabolism in Rheumatic Mitral Valve Disease
Source: PLoS One. 2014 Mar 6;9(3):e90527. doi: 10.1371/journal.pone.0090527 (PMC3948343; doi:10.1371/journal.pone.0090527)

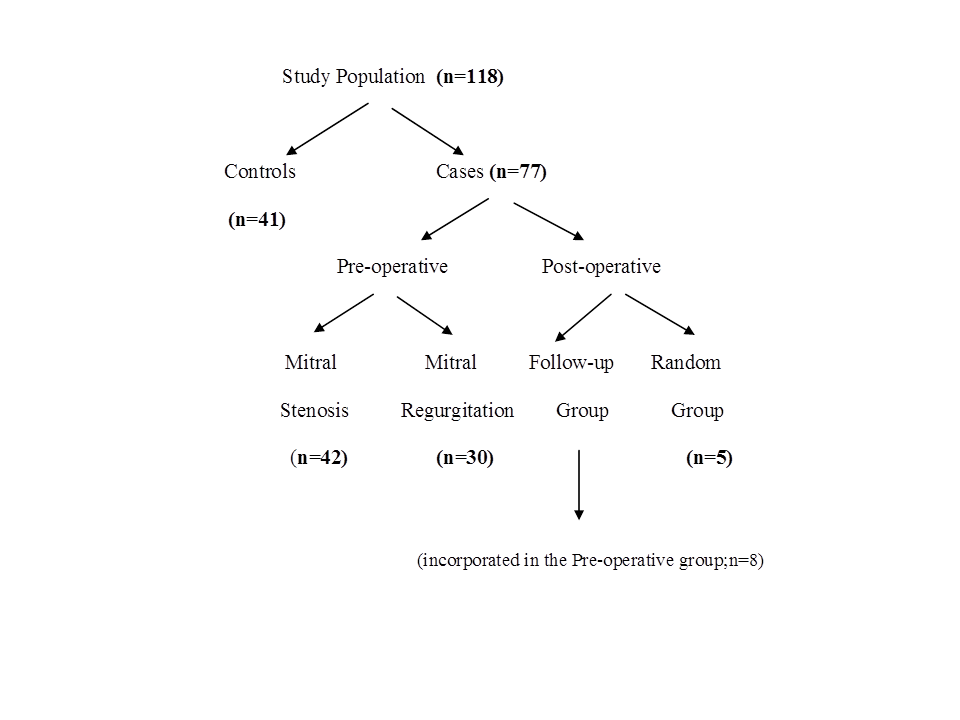

Supplement: Figure S1 — Flow Chart of Study Design. (TIF) [file pone.0090527.s001.tif]

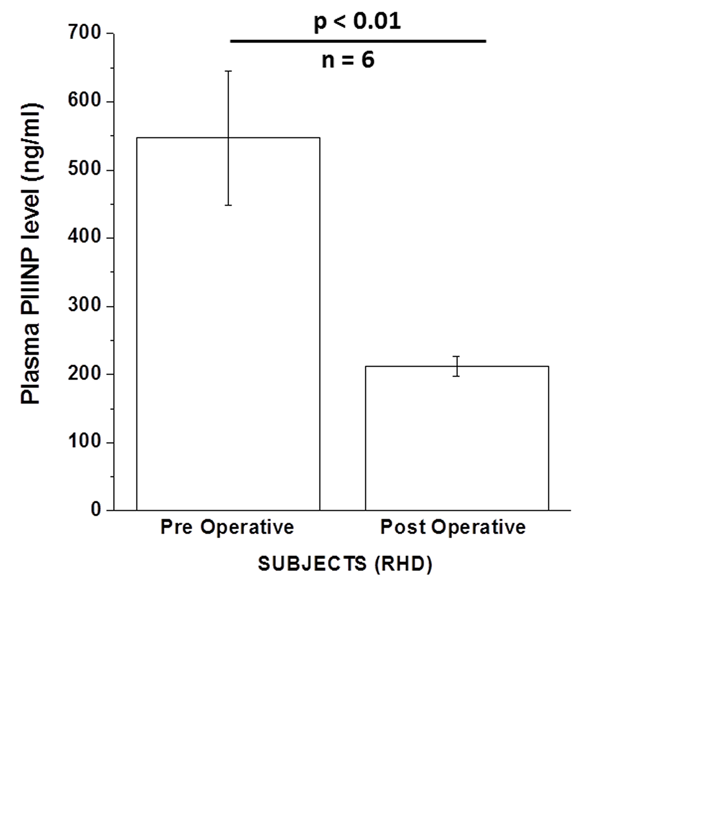

Supplement: Figure S2 — Comparison of plasma PIIINP level of the same RHD subjects (n = 6) before surgery and one month after mitral valve replacement. (TIF) [file pone.0090527.s002.tif]
