# Supplementary material for: Clinical Significance of Markers of Collagen Metabolism in Rheumatic Mitral Valve Disease
Source: PLoS One. 2014 Mar 6;9(3):e90527. doi: 10.1371/journal.pone.0090527 (PMC3948343; doi:10.1371/journal.pone.0090527)
Supplement: File S1 — Table S1. Characteristics of randomly collected post-operative RHD subjects. Table S2. Preoperative Echocardiographic Characteristics of randomly collected post-operative RHD subjects. Table S3 Level of collagen metabolism markers in male and female subjects with Mitral Stenosis and Mitral Regurgitation lesions. Table S4 Level of collagen metabolism markers in subjects with and without Atrial Fibrillation in Mitral Stenosis and Mitral Regurgitation groups. Table S5 Association of collagen metabolism markers with continuously distributed variables in subjects with Mitral Stenosis. Table S6. Association of collagen metabolism markers with continuously distributed variables in subjects with Mitral Regurgitation. (DOC) [file pone.0090527.s003.doc]

**Supporting Information (Tables)**

**Table S1.** Characteristics of randomly collected post-operative RHD subjects

| **Serial No.** | **Age** | **Sex** | **H/o RF** | **Pulse** | **SBP** | **DBP** | **Time of sample collection** | **Preoperative Echocardiographic Diagnosis** |
| --- | --- | --- | --- | --- | --- | --- | --- | --- |
|  |  |  |  |  |  |  |  |  |
| 1 | 48 | F | Y | 80 | 120 | 80 | More than 1 year after mitral valve replacement | Predominantly MS |
| 2 | 42 | M | Y | 80 | 150 | 90 | One month after mitral valve replacement | Predominantly MS |
| 3 | 48 | M | Y | 80 | 110 | 80 | One year after mitral valve replacement | Predominantly MS |
| 4 | 35 | F | N | 80 | 120 | 70 | One month after mitral valve replacement | Predominantly MR |
| 5 | 40 | F | Y | 84 | 110 | 70 | More than 1 year after mitral valve replacement | Predominantly MR |

DBP Diastolic Blood Pressure; F Female; M Male; MR Mitral Regurgitation; MS Mitral Stenosis; SBP Systolic Blood Pressure; Y Yes; N No

**Table S2. Preoperative Echocardiographic Characteristics of randomly collected post-operative RHD subjects**

| **Sl.No.** | **LA**  **(mm)** | **LVIDd**  **(mm)** | **EF%** | **PASP** | **MVA**  **(sq.mm)** | **LVIDs**  **(mm)** | **LVPW**  **(mm)** | **IVSD**  **(mm)** | **FS%** | **RWT** | **LVM**  **(Kg)** |
| --- | --- | --- | --- | --- | --- | --- | --- | --- | --- | --- | --- |
| **1** | - | 38 | 73 | - | - | 22 | 6 | 6 | 42 | 0.32 | 0.06 |
| **2** | 44 | 44 | 58 | Y | 70 | 27 | 9 | 9 | 39 | 0.41 | 0.13 |
| **3** | 44 | 45.8 | 52 | Y | 100 | 33.1 | 9 | 11.3 | 28 | 0.39 | 0.16 |
| **4** | 54.2 | 54 | 60 | - | - | 34 | 11 | 11 | 37 | 0.41 | 0.24 |
| **5** | - | 52 | 60 | - | 150 | 39.5 | - | - | 24 | **-** | **-** |

EF ejection fraction; FS fractional shortening; LA left atrium; LVIDd left ventricular internal diameter diastolic; LVIDs left ventricular internal diameter systolic; LVM Left Ventricular Mass; LVPW left ventricular posterior wall; MVA mitral valve area; PASP pulmonary artery systolic pressure; RWT relative wall thickness; Sl.No. serial number.

**Table S3** Level of collagen metabolism markers in male and female subjects with Mitral Stenosis and Mitral Regurgitation lesions

| **Marker** | **MS** | | | **MR** | | |
| --- | --- | --- | --- | --- | --- | --- |
|  | **Male** | **Female** | **p value** | **Male** | **Female** | **p value** |
|  |  |  |  |  |  |  |
|  |  |  |  |  |  |  |
| **PICP**  **(ng/ml)** | 1124 ± 188  (n=20) | 1415 ± 161  (n=19) | 0.25 | 690 ± 112  (n=7) | 903 ± 91  (n=20) | 0.22 |
|  |  |  |  |  |  |  |
| **PIIINP**  **(ng/ml)** | 514 ± 62  (n=17) | 567 ± 59  (n=16) | 0.54 | 494 ± 112  (n=5) | 584 ± 55  (n=20) | 0.47 |
|  |  |  |  |  |  |  |
| **MMP-1(ng/ml)** | 52 ± 13  (n=9) | 64 ± 16  (n=13) | 0.59 | 95 ± 28  (n=5) | 78 ± 16  (n=15) | 0.62 |
|  |  |  |  |  |  |  |
| **TIMP-1(ng/ml)** | 224 ± 53  (n=9) | 168 ± 36  (n=13) | 0.37 | 168 ± 46  (n=5) | 157 ± 24  (n=15) | 0.82 |
|  |  |  |  |  |  |  |
| **MMP-1/TIMP-1** | 0.42 ± 0.17  (n=9) | 0.46 ± 0.09  (n=13) | 0.80 | 0.64 ± 0.17  (n=5) | 0.53 ± 0.08  (n=15) | 0.51 |

|  |
| --- |

Values indicate mean ± SEM. MMP-1, matrix metalloproteinase -1; MS, mitral stenosis; MR, mitral regurgitation; n, number of subjects; PICP, carboxy terminal propeptide of type I collagen; PIIINP, amino terminal propeptide of type III collagen; TIMP-1, tissue inhibitor of matrix metalloproteinase-1.

**Table S4** Level of collagen metabolism markers in subjects with and without Atrial Fibrillation in Mitral Stenosis and Mitral Regurgitation groups

| **Marker** | **MS** | | | **MR** | | |
| --- | --- | --- | --- | --- | --- | --- |
|  | **AF** | **NO AF** | **p**  **value**  **va** | **AF** | **NO AF** | **p value** |
|  |  |  |  |  |  |  |
| **PICP**  **(ng/ml)** | 1325 ± 217  (n=13) | 1236 ± 156  (n=26) | 0.74 | 1087 ± 146  (n=8) | 748 ± 78  (n=19) | 0.03^*^ |
|  |  |  |  |  |  |  |
| **PIIINP**  **(ng/ml)** | 518 ± 56  (n=14) | 555 ± 62  (n=19) | 0.67 | 546 ± 68  (n=7) | 573 ± 63  (n=18) | 0.81 |
|  |  |  |  |  |  |  |
| **MMP-1(ng/ml)** | 58 ± 17  (n=8) | 59 ± 14  (n=14) | 0.97 | 74 ± 16  (n=6) | 86 ± 19  (n=14) | 0.72 |
|  |  |  |  |  |  |  |
| **TIMP-1(ng/ml)** | 276 ± 68  (n=8) | 143 ± 20  (n=14) | 0.03^*^ | 174 ± 97  (n=6) | 154 ± 23  (n=14) | 0.67 |
|  |  |  |  |  |  |  |
| **MMP-1/TIMP-1** | 0.33 ± 0.13  (n=8) | 0.51 ± 0.12  (n=14) | 0.34 | 0.51 ± 0.12  (n=6) | 0.58 ± 0.09  (n=14) | 0.66 |

|  |
| --- |

Values indicate mean ± SEM. AF, atrial fibrillation;MMP-1, matrix metalloproteinase -1; MS, mitral stenosis; MR, mitral regurgitation; n, number of subjects; PICP, carboxy terminal propeptide of type I collagen; PIIINP, amino terminal propeptide of type III collagen; TIMP-1, tissue inhibitor of matrix metalloproteinase-1. *p<0.05 between groups under comparison.

**Table S5** Association of collagen metabolism markers with continuously distributed variables in subjects with Mitral Stenosis

| **Parameter** | **Collagen Metabolism Markers** | | | | | | | | | |
| --- | --- | --- | --- | --- | --- | --- | --- | --- | --- | --- |
|  |  |  |  |  |  |  |  |  |  |  |
|  | **PICP**  **(ng/ml)** | | **PIIINP**  **(ng/ml)** | | **MMP-1 (ng/ml)** | | **TIMP-1 (ng/ml)** | | **MMP-1 /TIMP-1** | |
|  |  | |  | |  | |  | |  | |
|  | **r** | **p** | **r** | **p** | **r** | **p** | **r** | **p** | **r** | **p** |
| **Age(yrs.)**  **Pulse(bpm)**  **SBP(mm Hg)**  **DBP(mm Hg)**  **LA diameter**  **(mm)**  **LVIDd(mm)**  **LVIDs(mm)**  **LVPW(mm)**  **IVSD(mm)**  **EF(%)**  **PASP**  **(mm Hg)**  **MVA(sq.mm)**  **FS(%)**  **RWT**  **LV Mass(Kg)** | -0.24  -0.18  -0.24  -0.23  -0.13  -0.03  -0.01  -0.37  -0.39  -0.03 | 0.15  0.26  0.14  0.17  0.46  0.88  0.93  0.03^*^  0.02^*^  0.88 | 0.24  -0.14  -0.09  -0.06  -0.19  -0.28  -0.35  -0.09  -0.11  0.36 | 0.19  0.44  0.61  0.74  0.32  0.11  0.04^*^  0.62  0.56  0.04^*^ | 0.21  -0.13  -0.13  -0.08  -0.27  0.27  0.53  0.08  -0.17  -0.54  -0.15  0.31  -0.52  -0.04  0.08 | 0.34  0.56  0.57  0.71  0.23  0.23  0.01^*^  0.73  0.44  0.009^‡^  0.58  0.17  0.01^*^  0.85  0.74 | -0.16  -0.05  -0.09  -0.13  0.07  -0.22  -0.29  -0.03  0.23  0.21  0.32  -0.13  0.21  0.11  -0.10 | 0.48  0.83  0.70  0.57  0.75  0.33  0.19  0.90  0.30  0.35  0.23  0.56  0.35  0.64  0.65 | 0.25  -0.02  -0.15  -0.03  -0.26  0.30  0.55  0.14  -0.10  -0.44  ---  ---  -0.51  -0.04  0.19 | 0.27  0.92  0.49  0.88  0.26  0.17  0.008^‡^  0.54  0.65  0.04^*^  ---  ---  0.02^*^  0.86  0.40 |
|  | ---  ---  0.10  -0.29  -0.30 | ---  ---  0.54  0.08  0.07 | ---  ---  0.21  0.09  -0.27 | ---  ---  0.24  0.63  0.13 |  |  |  |  |  |  |
|  |  |  |  |  |  |  |  |  |  |  |

bpm , beats per minute; DBP, diastolic blood pressure; EF, ejection fraction; FS, fractional shortening; IVSD, inter-ventricular septal diameter; LA, left atrium; LVIDd, left ventricular internal diameter diastolic; LVIDs, left ventricular internal diameter systolic; LVPW, left ventricular posterior wall; MMP-1, matrix metalloproteinase -1;MVA, mitral valve area; PASP, pulmonary artery systolic pressure; PICP, carboxy terminal propeptide of type I collagen; PIIINP, amino terminal propeptide of type III collagen; r, Pearson’s correlation coefficient; RWT, relative wall thickness; SBP, systolic blood pressure; TIMP-1, tissue inhibitor of matrix metalloproteinase-1. ---, see figure 3; * , p<0.05 ; ‡ , p<0.01.

**Table S6. Association of collagen metabolism markers with continuously distributed variables in subjects with Mitral Regurgitation**

| **Parameter** | **Collagen Metabolism Markers** | | | | | | | | | |
| --- | --- | --- | --- | --- | --- | --- | --- | --- | --- | --- |
|  | **PICP**  **(ng/ml)** | | **PIIINP**  **(ng/ml)** | | **MMP-1 (ng/ml)** | | **TIMP-1 (ng/ml)** | | **MMP-1**  **/TIMP-1** | |
|  | **r** | **p** | **r** | **p** | **r** | **p** | **r** | **p** | **r** | **p** |
| **Age(yrs.)** | 0.18 | 0.36 | -0.11 | 0.59 | -0.31 | 0.18 | -0.05 | 0.83 | -0.25 | 0.29 |
| **Pulse(bpm)** | -0.29 | 0.15 | -0.17 | 0.41 | 0.007 | 0.98 | -0.11 | 0.64 | 0.13 | 0.58 |
| **SBP(mm Hg)** | -0.03 | 0.89 | -0.18 | 0.39 | -0.08 | 0.73 | 0.31 | 0.19 | -0.21 | 0.38 |
| **DBP(mm Hg)** | -0.004 | 0.99 | -0.05 | 0.82 | -0.12 | 0.62 | 0.27 | 0.25 | -0.24 | 0.31 |
| **LA diameter**  **(mm)** | 0.56 | 0.003^‡^ | 0.25 | 0.22 | 0.23 | 0.33 | 0.25 | 0.28 | 0.01 | 0.96 |
| **LVIDd(mm)** | --- | --- | --- | --- | 0.02 | 0.95 | 0.36 | 0.12 | --- | --- |
| **LVIDs(mm)** | --- | --- | --- | --- | -0.23 | 0.33 | 0.31 | 0.18 | --- | --- |
| **LVPW(mm)** | 0.30 | 0.13 | 0.2 | 0.34 | -0.008 | 0.97 | 0.12 | 0.6 | -0.23 | 0.32 |
| **IVSD(mm)** | 0.34 | 0.08 | 0.23 | 0.27 | -0.04 | 0.88 | 0.16 | 0.49 | -0.32 | 0.18 |
| **EF(%)** | -0.31 | 0.11 | 0.006 | 0.98 | -0.02 | 0.94 | -0.4 | 0.08 | 0.28 | 0.24 |
| **PASP**  **(mm Hg)** | -0.23 | 0.52 | 0.79 | 0.02**:** | 0.05 | NA | -0.10 | NA | 0.69 | 1.0 |
| **MVA(sq.mm)** | 0.45 | 0.20 | -0.06 | 0.88 | 0.54 | 0.17 | 0.26 | 0.54 | 0.36 | 0.38 |
| **FS(%)** | -0.16 | 0.43 | 0.18 | 0.40 | 0.27 | 0.25 | -0.11 | 0.63 | 0.27 | 0.26 |
| **RWT** | -0.39 | 0.04**:** | -0.10 | 0.64 | -0.05 | 0.84 | -0.15 | 0.53 | 0.09 | 0.71 |
| **LV Mass(Kg)** | --- | --- | --- | --- | 0.013 | 0.96 | 0.34 | 0.14 | --- | --- |

bpm, beats per minute; DBP, diastolic blood pressure; EF, ejection fraction; FS, fractional shortening; IVSD, inter-ventricular septal diameter; LA, left atrium; LVIDd, left ventricular internal diameter diastolic; LVIDs, left ventricular internal diameter systolic; LVPW, left ventricular posterior wall; MMP-1, matrix metalloproteinase -1;MVA, mitral valve area; PASP, pulmonary artery systolic pressure; PICP, carboxy terminal propeptide of type I collagen; PIIINP, amino terminal propeptide of type III collagen; r, Pearson’s correlation coefficient; RWT, relative wall thickness; SBP, systolic blood pressure; TIMP-1, tissue inhibitor of matrix metalloproteinase-1; ---, see figure 4; : , p<0.05 ; ‡, p<0.01.
